# Is There an Interplay between Immune Checkpoint Inhibitors, Thromboprophylactic Treatments and Thromboembolic Events? Mechanisms and Impact in Non-Small Cell Lung Cancer Patients

**DOI:** 10.3390/cancers12010067

**Published:** 2019-12-25

**Authors:** Federico Nichetti, Francesca Ligorio, Emma Zattarin, Diego Signorelli, Arsela Prelaj, Claudia Proto, Giulia Galli, Antonio Marra, Giulia Apollonio, Luca Porcu, Filippo de Braud, Giuseppe Lo Russo, Roberto Ferrara, Marina Chiara Garassino

**Affiliations:** 1 Department of Medical Oncology, Fondazione IRCCS Istituto Nazionale dei Tumori, 20133 Milan, Italy; francesca.ligorio@istitutotumori.mi.it (F.L.); emma.zattarin@istitutotumori.mi.it (E.Z.); Diego.Signorelli@istitutotumori.mi.it (D.S.); Arsela.Prelaj@istitutotumori.mi.it (A.P.); Claudia.proto@istitutotumori.mi.it (C.P.); giulia.galli@istitutotumori.mi.it (G.G.); Giulia.Apollonio@istitutotumori.mi.it (G.A.); Filippo.DeBraud@istitutotumori.mi.it (F.d.B.); Giuseppe.LoRusso@istitutotumori.mi.it (G.L.R.); Roberto.Ferrara@istitutotumori.mi.it (R.F.); Marina.Garassino@istitutotumori.mi.it (M.C.G.); 2 Department of Medical Oncology, Istituto Europeo di Oncologia, 20141 Milan, Italy; Antonio.marra@ieo.it; 3 Department of Oncology, IRCCS Istituto di Ricerche Farmacologiche Mario Negri, 20156 Milan, Italy; luca.porcu@marionegri.it; 4 Department of Oncology and Hemato-Oncology, University of Milan, 20122 Milan, Italy

**Keywords:** immunotherapy, immune checkpoint inhibitors, thromboprophylaxis, aspirin, thromboembolic events, non-small cell lung cancer

## Abstract

PD-1 pathway blockade has been shown to promote proatherogenic T-cell responses and destabilization of atherosclerotic plaques. Moreover, preclinical evidence suggests a potential synergy of antiplatelet drugs with immune checkpoint inhibitors (ICIs). We conducted an analysis within a prospective observational protocol (APOLLO study) to investigate the rates, predictors, and prognostic significance of thromboembolic events (TE) and thromboprophylaxis in patients with advanced NSCLC treated with ICIs. Among 217 patients treated between April 2014 and September 2018, 13.8% developed TE events. Current smoking status (HR 3.61 (95% CI 1.52–8.60), *p* = 0.004) and high (>50%) PD-L1 (HR 2.55 (95% CI 1.05–6.19), *p* = 0.038) resulted in being independent TE predictors. An increased risk of death following a diagnosis of TE (HR 2.93; 95% CI 1.59–5.42; *p* = 0.0006) was observed. Patients receiving antiplatelet treatment experienced longer progression-free survival (PFS) (6.4 vs. 3.4 months, HR 0.67 (95% CI 0.48–0.92), *p* = 0.015) and a trend toward better OS (11.2 vs. 9.6 months, HR 0.78 (95% CI 0.55–1.09), *p* = 0.14), which were not confirmed in a multivariate model. No impact of anticoagulant treatment on patients’ outcomes was observed. NSCLC patients treated with ICIs bear a consistent risk for thrombotic complications, with a detrimental effect on survival. The impact of antiplatelet drugs on ICIs efficacy deserves further investigation in prospective trials.

## 1. Introduction

Non-small cell lung cancer (NSCLC) is the leading cause of cancer related death worldwide, with a five year overall survival rate of 10–15% [1]. Thromboembolic (TE) events represent a major complication among NSCLC patients, being reported in up to 14% of cases [2]. Among solid cancers, NSCLC patients are at higher risk of TE events, particularly during anticancer chemotherapy, resulting in worsened quality of life, increased health care costs, and shorter survival [2,3].

In recent years, the advent of immunotherapy has revolutionized the treatment landscape of NSCLC. Several clinical trials demonstrated the superior efficacy of immune checkpoint inhibitors (ICIs) over chemotherapy in the first and more advanced lines of treatment [4,5,6,7,8,9,10], with a markedly different toxicity profile. Monoclonal antibodies targeting the programmed cell death 1 (PD-1) and the programmed cell death ligand 1 (PD-L1) axis have been associated with a spectrum of immune related adverse events (irAEs), arising as a results of an aberrant activation of the immune system, thus leaving common chemotherapy associated AEs in the background [11]. Among these, TE events have not been commonly considered as an ICIs drug related toxicity and extremely low rates have been reported in ICI clinical trials, ranging from 0 to less than 5% [4,5,6,7,8,9,10]. A recent meta-analysis analyzing single agent PD-1/PD-L1 inhibitors and irAEs across different cancer types did not include thromboembolism as a toxicity outcome, with only single patient cases of TE events reported as treatment related deaths [12]. For these reasons, the incidence of TE events and potential variables able to identify patients at higher risk in this population are currently undefined [13]. Nevertheless, PD-1 pathway blockade has been shown to promote proatherogenic T-cell responses and growth and destabilization of atherosclerotic plaques in mouse models, suggesting that ICIs could potentially foster arterial vascular events [14,15]. Furthermore, despite a partially different pathogenesis, there is an emerging consensus that that venous and arterial TE events are not quite as disparate as previously thought and should be both investigated as a cancer related complication [16].

Given the negative impact of TE events on morbidity and mortality, anticoagulant and antiplatelet agents have been investigated as potential strategies to improve cancer patients’ outcome. Experimental evidence suggests that low molecular weight heparin (LMWH) might have antitumor effects via inhibition of angiogenesis and metastatic spread [17]. However, clinical studies evaluating LMWH in lung cancer patients provided modest results [18,19,20], so that thromboprophylaxis is not routinely recommended in clinical practice. Considering antiplatelet drugs, preclinical data suggest that cyclooxygenase (COX) driven prostaglandin E2 (PGE2) might promote tumor immune escape and regulate PD-L1 expression within the tumor microenvironment [21,22,23,24]. Moreover, COX inhibitors (COXi) such as aspirin (ASA) and celecoxib significantly synergized with anti-PD-1 treatment in mouse models of melanoma and colorectal cancer [21,25]. While the addition of COXi to standard chemotherapy failed to improve patient outcomes in NSCLC [26,27,28], their use was associated with longer time to progression in ICI treated melanoma patients [29], and no data in NSCLC patients are currently available. COX-1/-2 and the microsomal form of human prostaglandin E synthase 1 (mPGES1) are both known to be overexpressed in NSCLC cells, suggesting a potential role for COXi in enhancing ICIs’ efficacy also in this scenario [30].

In this study, we aimed at exploring the impact of TE events and anticoagulant and antiplatelet treatments in patients with NSCLC treated with ICIs.

## 2. Materials and Methods

### 2.1. Study Population

The present study was an unplanned analysis within a prospective observational protocol approved by the local Institutional Review Board (INT 22_15, APOLLO study) and was conducted in accordance with the Declaration of Helsinki. Medical records of all consecutive patients with locally advanced or metastatic NSCLC who completed at least one treatment cycle with ICIs, at Istituto Nazionale dei Tumori, Milan, Italy, between April 2014 and September 2018 were retrospectively reviewed. All patients signed a written informed consent. ICIs included PD-1 inhibitors (nivolumab and pembrolizumab), PD-L1 inhibitors (atezolizumab, avelumab, and durvalumab), and cytotoxic T-lymphocyte antigen 4 (CTLA-4) inhibitors (ipilimumab and tremelimumab). Treatments were provided until disease progression, unacceptable toxicity, or consent withdrawal. All patients were followed up until death, loss of contact, or time of data lock (31 January 2019). Treatment response was assessed per Response Evaluation Criteria in Solid Tumors (RECIST) v1.1.

### 2.2. Identification of TE Events and Anticoagulant and Antiplatelet Treatment

Both venous (deep venous thrombosis of the limbs, pulmonary embolism, or venous thrombosis of other districts excluding superficial thrombophlebitis) and arterial (acute coronary syndrome, cerebrovascular accident, or arterial thrombosis of other districts) TE events were identified by retrospective review of medical records including diagnostic imaging results (computerized tomography (CT), CT angiography, doppler ultrasonography), medication history, and review of outside medical records when available. Asymptomatic TE reported as an incidental imaging finding was included as a TE event. TE events were included while on ICI therapy or if they occurred within 90 days after ICI suspension and no other cancer treatment had been initiated by the patient. TE occurring during subsequent treatment lines were not considered as events. Baseline data regarding previous TE events and ongoing prophylactic or therapeutic anticoagulant treatment and antiplatelet treatment were also recorded. For the purpose of the study, patients were defined as receiving thromboprophylaxis if anticoagulant or antiplatelet treatment was ongoing at ICI treatment initiation and was not suspended before the first disease assessment.

### 2.3. Study Objectives

The main objectives of the study were (1) to evaluate the rates of TE events in NSCLC patients treated with ICIs and (2) to explore the impact of TE events and anticoagulant and antiplatelet treatments on patients’ survival. Secondary objectives were to investigate potential risk factors for TE events and to assess the risk assessment capacity of the Khorana score in this population [31].

### 2.4. Statistical Analysis

Descriptive statistics were used to analyze and report patients’ characteristics. Association between categorical or continuous variables and TE events were performed by the Chi-squared or Wilcoxon–Mann–Whitney test, respectively. The thresholds for neutrophil to lymphocyte ratio (NLR) (outside the lung immune prognostic index (LIPI)) and platelet to lymphocyte ratio (PLR) were determined by calculating the area under the curve from the receiver operating characteristic (ROC) curves according to TE event occurrence [32]. Progression-free survival (PFS) was calculated from ICI start to the date of radiological or clinical disease progression, last follow-up, or death for any cause. Overall survival (OS) was calculated from ICI start to the date of death or last follow-up. Survival curves were estimated by the Kaplan–Meier method and compared by log-rank. Duration of follow-up was calculated using the reverse Kaplan–Meier method. To assess the impact of TE events on survival, OS of patients who developed TE events was compared to those who did not. To eliminate the likelihood of immortal time bias (due to the time dependent nature of TE) [33], TE events were tested as a time varying covariate, where patients started in the “no TE event” group and were switched to the “TE event” group at the date of TE diagnosis. To test the biological relationship between TE events and covariates, TE event specific hazards were calculated considering the interval from the date of ICI start to the first date of TE identification, and patients not developing TE before starting any subsequent anticancer treatment were censored at the last date of follow-up or death. Cox’s proportional hazards models were used to identify the strongest predictors of TE development and for survival analyses. Hazards ratios (HRs) together with 95% confidence intervals (CI) were provided. Multivariate analysis was calculated for the significant (*p* ≤ 0.1) variables by the univariate test or by a priori selection for biological relevance. The statistical significance threshold was set to a two tailed 0.05 value. R software (Version 3.5.3) and RStudio software (Version 1.1.456) were used for statistical analyses.

## 3. Results

### 3.1. Patients’ Characteristics

A total of 217 patients were included. At the moment of data analysis, 30 patients (13.8%) developed TE events, 181 (83.4%) had progressed, and 166 (76.5%) had died. Median follow up was 37.8 (22.6–43.9) months. Patients’ characteristics of the entire study population and according to occurrence of TE events are shown in Table 1. Baseline laboratory values are provided in Table S1. No significant differences in terms of clinical and biological characteristics were observed between patients experiencing TE events or not except for smoking status and PD-L1 expression. Specifically, the percentages of current smokers (42.9% vs. 23.3%, *p* = 0.05) and of patients with tumor PDL-1 expression >50% (43.3 vs. 18.8%, *p* = 0.01) were significantly higher among the TE event group compared to the no TE event group. Regarding blood parameters, TE events occurred more frequently in patients with lower baseline PLR (*p* = 0.002) and lower NLR (*p* = 0.053), with a threshold defined by ROC curves of 181 and 3.2, respectively.

Treatment characteristics are reported in Table 2. The majority of patients (151, 69.6%) underwent treatment with anti-PD1 (nivolumab in 117 cases, pembrolizumab in 34 cases), 58 (26.7%) with an anti-PD-L1 (atezolizumab in 16 cases, avelumab in 4 cases, and durvalumab in 38 cases), and 8 (3.7%) patients with combined durvalumab + tremelimumab. The median number of administered treatment cycles and treatment duration were markedly higher in the TE group (20 (9–31) vs. 6 (3–16) cycles (*p* < 0.001) and 9.4 (5.4–21.7) vs. 2.9 (1.4–9.0) months (*p* < 0.001), respectively). ICI treatment was still ongoing at the time of database lock in 31 cases (14.3%). The overall objective response rate was 18.9%, whereas the disease control rate was 54.8% and both were significantly higher in patients experiencing TE events (*p* = 0.015 and 0.001, respectively). No significant differences in terms of irAEs were observed between the two groups. 

### 3.2. Clinical Characteristics and Risk Factors of TE Events

**Figure 1:**
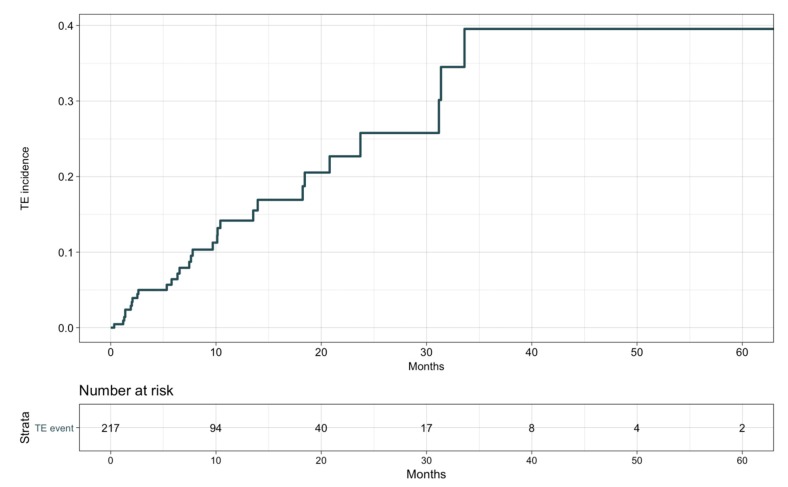
Thromboembolic events’ cumulative incidence.

The detailed description of TE events is provided in Table S2. Thirty (13.8%) patients developed TE events, with 16 venous (5 deep vein thrombosis, 6 pulmonary embolism, 2 portal vein thrombosis, 3 miscellaneous) and 14 arterial (2 acute coronary syndromes, 9 strokes, 3 visceral arterial thromboses) cases. Two venous TE events occurred after definitive ICI suspension for disease progression, but before any other treatment was initiated. Median time to occurrence of TE events was 7.5 months (range 1.2–33.6 months; see Figure 1), with no difference between arterial and venous cases. TE events were never the cause of treatment suspension or death in our population. No patient had more than one TE event during ICIs.

In the whole study population, sixty-nine patients (31.8%) were on antiplatelet treatment, of whom 61 were ASA users, the remaining taking clopidogrel or ticlopidine. Forty-seven (21.7%) patients were under anticoagulant treatment, of whom 44 with LMWH and 8 receiving a therapeutic dosage. No significant difference was observed for anticoagulant or antiplatelet treatment use in TE patients compared to no TE patients.

The Cox regression analysis identified variables affecting the risk of TE events (Table 3): by univariate analysis, the best predictors of TE events were smoking status (HR 2.48; 95% CI 1.17–5.26; *p* = 0.02) and PD-L1 positivity (HR 2.16; 95% CI 0.94–4.95; *p* = 0.07), while high PLR (HR 0.42; 95% CI 0.19–0.92; *p* = 0.03) and presence of >2 metastatic sites (HR 0.52; 95% CI 0.24–1.12; *p* = 0.09) were associated with a reduced TE risk. Kaplan–Meier curves for TE event specific hazards according to these variables are available in Figures S1–S4. Given the number of target events and the biological rationale, only smoking status, PD-L1 status, and PLR were included in the multivariate analysis (Table 4). This model confirmed an independent association between smoking status (HR 3.61; 95% CI 1.52–8.60; *p* = 0.004), PD-L1 positive status (HR 2.55; 95% CI 1.05–6.19; *p* = 0.038), and TE events. Of note, the Khorana score showed only a non-significant trend for TE prediction, and anticoagulant or antiplatelet treatments did not result in a protective effect (Table 3).

### 3.3. Impact of TE Events and Anticoagulant and Antiplatelet Treatments on Survival

**Figure 2:**
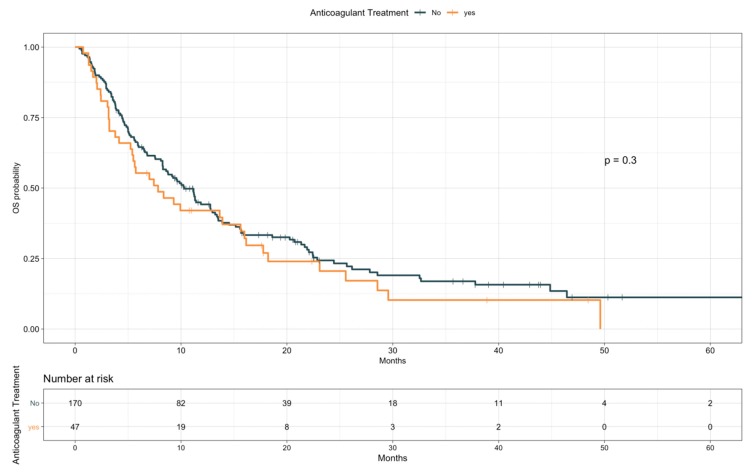
Overall survival according to anticoagulant treatment.

**Figure 3:**
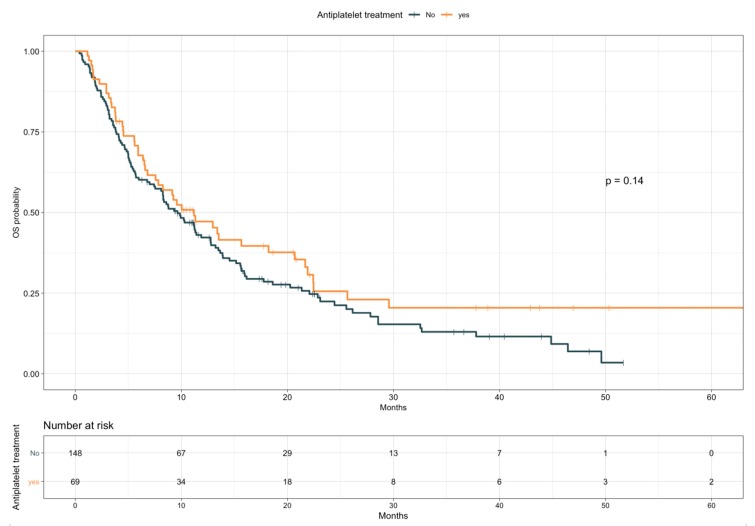
Overall survival according to antiplatelet treatment.

The impact of TE events and anticoagulant and antiplatelet treatments on patients’ OS was first assessed by univariate analysis (Table 5). Given the preliminary associations with a better objective response rate, disease control rate, and treatment duration, the effect of TE diagnosis on OS was first explored as a time independent variable. Patients experiencing TE events showed longer OS compared with those who did not (median, 15.9 vs. 8.8 months, HR 0.59; 95% CI, 0.37–0.93; *p* = 0.02; see Figure S5). However, with TE events defined as a time varying covariate, we observed an increased risk of death following a diagnosis of TE, with an overall univariable HR of 1.70 (95% CI, 1.06–2.72; *p* = 0.029). No significant impact on OS was detected for anticoagulant treatment (median, 7.8 vs. 10.3 months, HR 1.21; 95% CI, 0.84–1.73; *p* = 0.31; Figure 2), considered either prophylactic or therapeutic, nor for antiplatelet therapy, despite an observed favorable trend (median, 11.2 vs. 9.6 months, HR 0.78; 95% CI 0.55–1.09; *p* = 0.14; Figure 3). A multivariate model including other covariates statistically associated with OS (i.e., ECOG PS, PD-L1 status, line of treatment, and LIPI) was performed. Since NLR and PLR positively correlated with each other (Pearson coefficient correlation = 0.68; *p* < 0.001) and NLR was included in the LIPI, PLR was excluded from the multivariate analysis. In this model, TE events kept an independent negative prognostic value in terms of OS (HR 2.93; 95% CI 1.59–5.42; *p* = 0.0006; Table 6).

**Figure 4:**
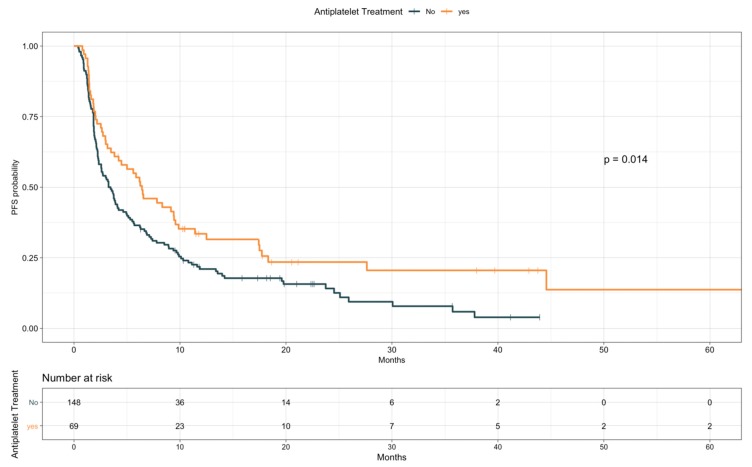
Progression-free survival according to antiplatelet treatment.

**Figure 5:**
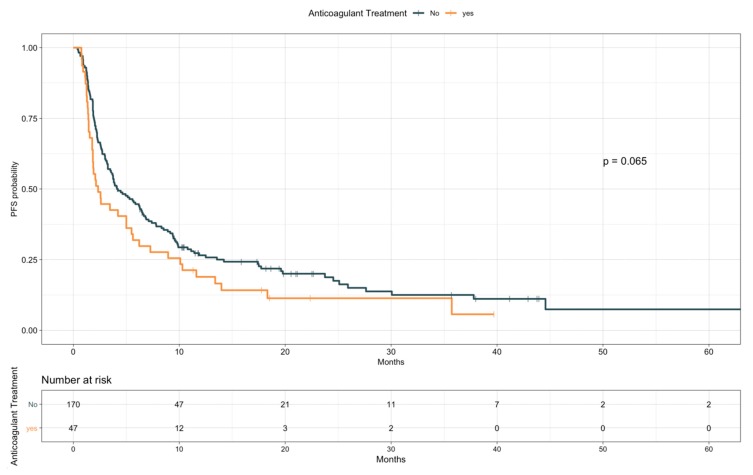
Progression-free survival according to anticoagulant treatment.

When looking at PFS (Table 7), antiplatelet drugs users were characterized by a longer PFS compared to nonusers (median, 6.4 vs. 3.4 months, HR 0.67; 95% CI 0.48–0.92; *p* = 0.015; Figure 4). Notably, a comparable difference was observed when restricting the analysis only to patients taking ASA (median, 6.5 vs. 3.4 months, HR 0.67; 95% CI 0.48–0.94; *p* = 0.021; Figure S6). Conversely, baseline anticoagulants use resulted in a trend for worse PFS (median, 2.3 vs. 4.2 months, HR 1.38; 95% CI 0.98–1.96; *p* = 0.065; Figure 5). Patients receiving anticoagulant treatment had significantly worse baseline ECOG PS (see Table S3), which could explain the lower PFS experienced upon ICIs. In the multivariate model including other covariates statistically associated with PFS (i.e., ECOG PS, PD-L1 status, presence of liver metastases, line of treatment, and LIPI), antiplatelet and anticoagulant treatments did not confirm an effect on patients PFS (HR 0.96; 95% CI, 0.59–1.57; *p* = 0.88, and HR 1.38; 95% CI, 0.82–2.32; *p* = 0.22, respectively; Table 8).

## 4. Discussion

Thromboembolic events are a common complication in patients with lung cancer, contributing to increased morbidity and mortality [34,35]. To date, the incidence of TE events in cancer patients receiving immunotherapy is unknown. Preclinical data showed that the PD-1/PD-L1 pathway plays an important role in downregulating proatherogenic T-cell responses, so that its blockade may lead to increased levels of pro-inflammatory cytokines and T-cell driven progression and destabilization of atherosclerotic plaques [14,15]. In the clinical setting, only a few isolated reports described thrombotic events as possible irAEs, mostly as arterial cases [36,37,38]. Tomita et al. presented a first report of acute coronary syndrome in a PD-L1 high, NSCLC patient who achieved complete response with nivolumab, suggesting it as a possible immune related adverse event [37]. Boutros and colleagues described four TE cases during ICI treatment, highlighting the presence of large amounts of entrapped leucocytes in a patient’s thrombus after embolectomy [38]. Finally, in a large series including patients with different cancer subtypes receiving ICIs, an unexpected high rate of TE events (30.3%) was reported, with a potential association with worsened survival [39].

In the present study, we described for the first time the rates and characteristics of TE events in NSCLC patients receiving ICIs. We showed that the risk is not negligible, as the percentage of patients developing thrombosis was in line with previous literature data in the overall NSCLC patients’ population [2]. Moreover, while TE risk is particularly high in the first few months following the start of chemotherapy [40], here we showed that TE events occurred throughout the course of ICI treatment, with two thirds of cases reported after the first six months. Of note, about half of the cases were represented by arterial events. In our opinion, these data raise two hypotheses: ICIs may directly increase the risk of arterial events (as suggested by mouse models) or these events may be linked to risk factors independent of patients’ cancer treatment. In this latter scenario, ICI long responders develop TE events as a result of longer survival, longer exposure to “other-than-cancer” risk factors (e.g., lifestyle, comorbidities), and thus longer time at risk for incident TE. In our series, the increased TE risk associated with smoking status and high PD-L1 expression agreed with both of these hypotheses, as these factors may promote atherogenesis, but also contribute to better patient outcome during ICI treatment. In particular, PGE2 concentration in lung cancer tissue significantly correlates with PD-1 expression on CD8^+^ tumor infiltrating lymphocytes, suggesting PGE2 related inflammation as a possible link between TE events and T-cells’ exhaustion [41]. Furthermore, we observed that the development of TE events during ICIs is an independent prognostic factor associated with worse survival when considered as a time dependent variable [39].

Overall, these observations raise the problem of how to predict and prevent TE events effectively in this population. Most risk assessment models for cancer associated thrombosis were developed on patients treated with chemotherapy and only for venous events [31,42], justifying why we did not observe a good prediction power for the Khorana score in our series. 

Regarding thromboprophylaxis, randomized trials have shown that LMWH and direct oral anticoagulants approximately halve the risk of venous TE events in cancer patients [43,44,45]. However, most studies did not consider arterial events and did not include a relevant number of patients treated with ICIs. In our study, we did not observe a significant difference in TE rates according to anticoagulant or antiplatelet treatment. Moreover, LMWH users experienced worse PFS and a trend toward worse OS. Due to the lack of rationale for a detrimental effect of LMWH on ICIs’ efficacy, we hypothesize that thromboprophylaxis was prescribed in patients in worse clinical conditions, with higher disease burden and reduced mobility.

Intriguingly, patients receiving antiplatelet treatment, and in particular ASA users, experienced longer PFS and a non-significant trend toward better OS. This benefit might also be underestimated in our series, since patients might have been receiving antiplatelet drugs for multiple comorbidities (e.g., hypertension, diabetes, previous cardiovascular accidents; see Table S4) that represent negative prognostic indicators themselves. Preclinical studies showed that the COX-2/mPGES1 pathway is able to promote tumor immune escape mechanisms by increasing the number of T regulatory cells [46] and myeloid derived suppressor cells [47], as well as by driving increased expression of indoleamine 2,3-dioxygenase 1 [48] and PD-L1 [24,49,50] by cancer cells. Moreover, a potential synergic effect between COX inhibition and anti-PD/PD-L1 immunotherapy has been demonstrated in mouse tumor models [21,22]. Our findings support these hypotheses, showing for the first time in a clinical scenario that COX inhibition with ASA is associated with better outcome in NSCLC patients treated with ICIs. Of note, prospective, randomized trials testing COX inhibitors (NCT03638297, NCT03245489) and PGE2 receptor 4 antagonists (NCT03696212, NCT03658772) in combination with ICIs are ongoing [23]. However, the effect of COX-2/mPGES1 pathway inhibition on PD-L1 expression and the impact of this modulation on ICIs efficacy are still controversial. While some in vitro studies reported that COX-2 inhibition reduced the expression of PD-L1 [24,49,51] and may potentially reduce exhaustion of T-cells in the tumor microenvironment [41], others highlighted that treatment with celecoxib did not affect PD-L1 levels in melanoma and NSCLC cells [22,52], and PD-L1 staining was also shown to be dramatically greater in mPGES1 knock out derived tumor tissues compared to controls [22]. Furthermore, the effect of other COXi (e.g., nonsteroidal anti-inflammatory drugs and paracetamol) on ICI treatment outcome might differ from that of low dose ASA due to the different COX selectivity and reversibility of the binding. Finally, an alternative way to reduce PGE2 through reprogramming the fatty acid metabolism of tumor infiltrating myeloid derived suppressor cells has been shown to synergize with PD-1 blockade in murine models [53].

Our study had clearly some limitations. In particular, the retrospective, monocentric nature of the investigation, the lack of a statistical power calculation, the limited number of events, the heterogeneity of treatment lines and ICIs administered (e.g., anti-PD-1 and anti-PD-L1), and the missing data like PD-L1 status for several cases might have affected our results. Likewise, we included patients receiving variable anticoagulant and antiplatelet compounds, with different treatment schedules (e.g., prophylactic or therapeutic dosages) and affecting different biological pathways (e.g., aspirin as a COX inhibitor vs. clopidogrel targeting P2Y_12_ on platelets). Future investigations should include prospective homogenous cohorts to prevent any bias on this issue. Despite a strong preclinical rationale, the initial association between antiplatelet agents’ use and ICIs’ efficacy was not confirmed when other factors were considered. A control arm (e.g., NSCLC patients treated with chemotherapy) with a propensity matching to compare the outcomes of patients with comparable baseline characteristics will be necessary to elucidate a potential synergy in future studies. Similarly, in order to demonstrate a direct correlation between ICIs and TE events, a control arm of patients with similar characteristics not receiving immunotherapy would have been optimal. However, this comparison is hard to perform, since NSCLC patients treated with chemotherapy are at even higher risk of TE events, as are patients with driver mutations receiving targeted therapies [54,55,56]. Moreover, if tested, lower rates of PD-L1 positivity would be found in these populations, as this might represent the reason to omit ICIs. Finally, given the partially different pathogenesis of arterial and venous TE events, future studies conducted on larger series analyzing these events separately could be useful.

## 5. Conclusions

In conclusion, we aimed at evaluating the clinical relevance of TE events and antithrombotic treatments in NSCLC patients treated with immunotherapy. To our knowledge, this is the first study to show to that these patients bear a consistent risk for thrombotic complications.

Our study was exploratory in its nature and clearly prevented definitive conclusions. Nevertheless, our data serve as a first report on this topic and deserve further investigation in prospective studies to better define patients at high risk for TE events as candidates for thromboprophylaxis. Our aim was to raise awareness on this issue, which might become even more significant as immunotherapy-chemotherapy combinations enter clinical practice in the near future [57,58].

Lastly, we investigated a potential association between the use of antiplatelet agents and ICI treatment outcomes. Together with previous preclinical evidence, our data provide an additional rationale to implement novel clinical trials testing COX-2 inhibitors’ potential to prevent NSCLC immune evasion, as well as to enhance the anti-tumor activity of PD-1/PD-L1 based immunotherapy. If these findings are confirmed, these compounds may represent a valid, low cost option to increase ICIs’ efficacy and patients’ outcomes.

## Figures and Tables

**Table 1:** Baseline patients’ characteristics in the whole case series and according to the presence or absence of TE events.

Characteristic	Overall *n* = 217	Without TE *n* = 187	With TE *n* = 30
Sex, Male	136 (62.7)	115 (61.5)	21 (70.0)
Age, median (range), y	70 (32–90)	70 (32–90)	70 (56–83)
≤65	77 (35.5)	66 (35.3)	11 (36.7)
>65	140 (64.5)	121 (64.7)	19 (63.3)
ECOG PS			
0–1	198 (91.2)	168 (89.8)	30 (100.0)
≥2	19 (8.8)	19 (10.2)	\
Smoking *			
Never or former	154 (71.0)	138 (76.7)	16 (57.1)
Current	54 (24.9)	42 (23.3)	12 (42.9)
Pathologic subtype			
Non-squamous	168 (77.4)	146 (78.1)	22 (73.3)
Squamous	49 (22.6)	41 (21.9)	8 (26.7)
Molecular status ^x^			
EGFR mutation	11 (5.1)	10 (6.3)	1 (3.7)
ALK translocation	2 (0.9)	1 (0.7)	1 (3.8)
PD-L1 status ^#^			
≤50%	89 (41.0)	79 (42.2)	10 (33.3)
>50%	48 (22.1)	35 (18.8)	13 (43.3)
Not assessed	80 (36.9)	73 (39.0)	7 (23.4)
Previous Tx with TKI	18 (8.3)	15 (8.0)	3 (10.0)
Disease stage			
Locally advanced	10 (4.6)	8 (4.3)	2 (6.7)
Metastatic	207 (95.4)	179 (95.7)	28 (93.3)
Number of disease sites **			
≤2	98 (45.2)	80 (42.8)	18 (60.0)
>2	109 (50.2)	99 (52.9)	10 (33.3)
Brain metastases	44 (20.3)	39 (20.9)	5 (16.7)
Liver metastases	44 (20.3)	39 (20.9)	5 (16.7)
Comorbidities			
Arterial hypertension	44 (20.3)	38 (20.3)	6 (20.0)
COPD	25 (11.5)	27 (11.8)	3 (10.0)
Diabetes mellitus	21 (9.7)	18 (9.6)	3 (10.0)
Previous ACS	18 (8.3)	13 (7.0)	5 (16.7)
Previous stroke	9 (4.1)	6 (3.2)	3 (10.0)
Atrial fibrillation	10 (4.6)	10 (5.4)	\
Previous venous TE events	37 (17.1)	31 (16.6)	6 (20.0)
BMI ^xx^			
>25	88 (40.6)	71 (47.3)	17 (68.0)
>30	41 (18.9)	34 (18.2)	7 (23.3)
Antiplatelet treatment	69 (31.8)	56 (29.9)	13 (43.3)
ASA based treatment	61 (28.1)	49 (26.2)	12 (40.0)
Anticoagulant treatment	47 (21.7)	42 (22.5)	5 (16.7)
Therapeutic dosage	8 (3.7)	7 (3.7)	1 (3.3)

Data are presented as *n* (%) except where otherwise noted. * Data were missing for 9 patients. ** Patients with locally advanced disease were excluded from the analysis. ^x^ No ROS1 rearrangements were detected. EGFR mutations and ALK rearrangements were not assessed in 31 and 39 patients, respectively. ^#^ Defined positive as a tumor proportion score (TPS) ≥50% using Dako clone 22C3 or Ventana clone SP263 antibodies. ^xx^ Data were missing for 42 patients. Abbreviations: ACS: acute coronary syndrome; ASA: aspirin; BMI: body mass index; COPD: chronic obstructive pulmonary disease; ECOG PS: Eastern Cooperative Oncology Group Performance Status; LMWH: low molecular weight heparin; TE events: thromboembolic events; TKI: tyrosine kinase inhibitor; Tx: treatment.

**Table 2:** Treatment characteristics in the whole case series and according to the presence or absence of TE events.

Characteristic	Overall *n* = 217	Without TE *n* = 187	With TE *n* = 30
Line of Treatment			
<2	55 (25.4)	51 (27.3)	4 (13.3)
≥2	162 (74.6)	136 (72.7)	26 (86.7)
Drug Class			
Anti-PD-1	151 (69.6)	133 (71.1)	18 (60.0)
Anti-PD-L1	58 (26.7)	46 (24.6)	12 (40.0)
Anti PD-L1 + Anti CTLA-4	8 (3.7)	8 (4.3)	\
No. of administered cycles, median (IQR)	7 (3–19)	6 (3–16)	20 (9–31)
Treatment duration, median (IQR, months)	3.6 (1.5–10.3)	2.9 (1.4–9.0)	9.4 (5.4–21.7)
Best Response			
Complete Response	5 (2.3)	4 (2.1)	1 (3.3)
Partial Response	36 (16.6)	26 (13.9)	10 (33.3)
Stable Disease	78 (35.9)	64 (34.2)	14 (46.7)
Progressive Disease	98 (45.2)	93 (49.7)	5 (16.7)
Objective Response	41 (18.9)	30 (16.0)	11 (36.7)
Disease Control	119 (54.8)	94 (50.3)	25 (83.3)
Adverse Events ≥2 (other than TE)			
Anemia	6 (2.8)	4 (2.1)	2 (6.7)
Thrombocytopenia	2 (0.9)	2 (1.1)	\
Fatigue	10 (4.6)	9 (4.8)	1 (3.3)
Liver toxicity	10 (4.6)	8 (4.3)	2 (6.7)
Arthralgia	4 (1.8)	4 (2.1)	\
Colitis/diarrhea	7 (3.2)	7 (3.7)	\
Hyper/hypothyroidism	9 (4.1)	7 (3.7)	2 (6.7)
Pneumonitis	9 (4.1)	8 (4.3)	1 (3.3)
Pruritus or skin rash	8 (3.7)	6 (3.2)	2 (6.7)

Data are presented as *n* (%) except where otherwise noted. Abbreviations: CTLA-4: cytotoxic T-lymphocyte antigen 4; IQR: interquartile range; PD-1/PD-L1: programmed death-1/programmed death-ligand 1; TE: thromboembolic events.

**Table 3:** Univariate analyses of TE event specific hazards according to patients and treatment characteristics.

Variables	Subgroups	HR	95% CI	*p*
Sex	Male vs. female	1.73	0.79–3.79	0.17
Age	>65 vs. ≤65	0.97	0.46–2.03	0.93
Smoking	Current vs. never/former	2.48	1.17–5.26	**0.02**
ECOG PS *	≥1 vs. 0	1.12	0.54–2.35	0.76
PD-L1 status	>50% vs. <50%	2.16	0.94–4.95	**0.07**
Pathologic subtype	Squamous vs. non-squamous	1.52	0.68–3.43	0.31
Number of disease sites	>2 vs. ≤2	0.52	0.24–1.12	**0.09**
Brain metastases	Yes vs. no	0.74	0.28–1.93	0.53
Liver metastases	Yes vs. no	0.74	0.28–1.93	0.53
BMI	>25 vs. ≤25	1.98	0.85–4.59	0.11
	>30 vs. ≤30	1.18	0.49–2.82	0.71
Previous venous TEs	Yes vs. no	1.12	0.46–2.75	0.80
Khorana score	2 vs. 1	1.37	0.58–3.20	0.47
	3 vs. 1	1.75	0.64–4.78	0.28
Antiplatelet treatment	Yes vs. no	1.57	0.76–3.25	0.22
Anticoagulant treatment	Yes vs. no	0.86	0.33–2.25	0.75
Drug class	Anti-PD-1 vs. anti-PD-L1 (+/−anti CTLA4)	1.23	0.59–2.55	0.59
Line of treatment	≥2 vs. <2	2.34	0.81–6.72	0.11
LDH	>480 vs. ≤480 U/L	2.01	0.85–4.73	0.11
NLR	>3.2 vs. ≤3.2	0.80	0.38–1.66	0.54
PLR	>181 vs. ≤181	0.42	0.19–0.92	**0.03**

The *p* value is indicated in bold numbers when statistically significant. * This threshold was set since no patients with baseline ECOG PS ≥ 2 had a TE event. Abbreviations: ASA: aspirin; BMI: body mass index; CI: confidence interval; LDH: lactate dehydrogenase; LMWH: low molecular weight heparin; NLR: neutrophil to lymphocyte ratio; PLR: platelet to lymphocyte ratio; ECOG PS: Eastern Cooperative Oncology Group Performance Status; TE: thromboembolic events.

**Table 4:** Multivariate analyses of TE event specific hazards according to patients and treatment characteristics.

Variables	Subgroups	HR	95% CI	*p*
Smoking	Current vs. never/former	3.61	1.52–8.60	**0.004**
PD-L1 status	>50% vs. <50%	2.55	1.05–6.19	**0.038**
PLR	> 181 vs. ≤181	0.53	0.21–1.33	0.178

The *p* value is indicated in bold numbers when statistically significant. Abbreviations: CI: confidence interval; PLR: platelet to lymphocyte ratio.

**Table 5:** Univariate Cox proportional hazards model for overall survival.

Variables	Subgroups	HR	95% CI	*p*
TE events	Yes vs. no, time independent	0.59	0.37–0.93	**0.023**
	Yes vs. no, time dependent	1.70	1.06–2.72	**0.029**
Anticoagulant treatment	Yes vs. no	1.21	0.84–1.73	0.31
Antiplatelet treatment	Yes vs. no	0.78	0.55–1.09	0.14
Sex	Male vs. female	1.26	0.92–1.73	0.16
Age	>65 vs. ≤65	1.06	0.77–1.45	0.74
Smoking	Current vs. never/former	0.89	0.62–1.28	0.53
ECOG PS	≥2 vs. 0–1	3.71	2.21–6.25	**<0.0001**
PD-L1 status	>50% vs. ≤50%	0.48	0.30–0.76	**0.0016**
Number of disease sites	>2 vs. ≤2	1.05	0.77–1.43	0.74
Brain metastases	Yes vs. no	1.04	0.71–1.51	0.85
Liver metastases	Yes vs. no	1.38	0.95–1.98	0.09
Line of treatment	≥2 vs. <2	1.63	1.09–2.43	**0.018**
BMI	>25 vs. ≤25	0.92	0.65–1.30	0.64
	>30 vs. ≤30	1.20	0.82–1.77	0.35
LIPI	High vs. low	3.44	2.11–5.62	**<0.0001**
	Intermediate vs. low	1.89	1.29–2.75	
PLR	311.18 vs. 145.73	1.17	1.05–1.31	**0.005**

The *p* value is indicated in bold numbers when statistically significant. Abbreviations: ASA: aspirin; BMI: body mass index; CI: confidence interval; LMWH: low molecular weight heparin; PLR: platelet to lymphocyte ratio; ECOG PS: Eastern Cooperative Oncology Group Performance Status; LIPI: lung immune prognostic index, TE events: thromboembolic events.

**Table 6:** Multivariate Cox proportional hazards models for overall survival.

Variables	Subgroups	HR	95% CI	*p*
TE events	Yes vs. no, time dependent	2.93	1.59–5.42	**0.0006**
ECOG PS	≥2 vs. 0–1	3.29	1.50–7.21	**0.0029**
PD-L1 status	>50% vs. ≤50%	0.36	0.21–0.64	**0.0004**
Line of treatment	≥2 vs. <2	1.64	0.93–2.91	0.0889
LIPI	High vs. low	3.14	1.58–6.27	**0.0001**
	Intermediate vs. low	3.14	1.82–5.40	

The *p* value is indicated in bold numbers when statistically significant. Abbreviations: ASA: aspirin; CI: confidence interval; ECOG PS: Eastern Cooperative Oncology Group Performance Status; LIPI: lung immune prognostic index.

**Table 7:** Univariate Cox proportional hazards model for progression-free survival.

Variables	Subgroups	HR	95% CI	*p*
Anticoagulant treatment	Yes vs. no	1.38	0.98–1.96	**0.065**
Antiplatelet treatment	Yes vs. no	0.67	0.48–0.92	**0.014**
Sex	Male vs. female	1.08	0.80–1.46	0.62
Age	>65 vs. ≤65	0.91	0.67–1.23	0.54
Smoking	Current vs. never/former	0.79	0.56–1.11	0.18
ECOG PS	≥2 vs. 0–1	2.55	1.57–4.13	**0.0001**
PD-L1 status	>50% vs. ≤50%	0.40	0.26–0.61	**<0.0001**
Number of disease sites	>2 vs. ≤2	1.00	0.75–1.35	0.99
Brain metastases	Yes vs. no	1.05	0.73–1.50	0.80
Liver metastases	Yes vs. no	1.44	1.02–2.05	**0.041**
Line of Treatment	≥2 vs. <2	1.78	1.23–2.57	**0.002**
BMI	>25 vs. ≤25	0.78	0.56–1.09	0.15
	>30 vs. ≤30	1.12	0.77–1.63	0.56
LIPI	High vs. low	2.80	1.76–4.47	**0.0001**
	Intermediate vs. low	1.59	1.11–2.26	
PLR	311.18 vs. 145.73	1.35	1.18–1.55	**<0.0001**

The *p* value is indicated in bold numbers when statistically significant. Abbreviations: ASA: aspirin; BMI: body mass index; CI: confidence interval; LMWH: low molecular weight heparin; PLR: platelet to lymphocyte ratio; ECOG PS: Eastern Cooperative Oncology Group Performance Status; LIPI: lung immune prognostic index.

**Table 8:** Multivariate Cox proportional hazards models for progression-free survival.

Variables	Subgroups	HR	95% CI	*p*
Antiplatelet treatment	Yes vs. no	0.96	0.59–1.57	0.88
Anticoagulant treatment	Yes vs. No	1.38	0.82–2.32	0.22
ECOG PS	≥2 vs. 0–1	2.01	0.94–4.32	0.073
Liver metastases	Yes vs. no	1.10	0.64–1.89	0.73
PD-L1 status	>50% vs. ≤50%	0.41	0.25–0.67	**0.0004**
Line of treatment	≥2 vs. <2	1.79	1.07–2.98	**0.025**
LIPI	High vs. low	2.74	1.41–5.31	**0.0039**
	Intermediate vs. low	2.04	1.25–3.32	

The *p* value is indicated in bold numbers when statistically significant. Abbreviations: ASA: aspirin; BMI: body mass index; CI: confidence interval; PLR: derived platelet to lymphocyte ratio; ECOG PS: Eastern Cooperative Oncology Group Performance Status; LIPI: lung immune prognostic index.

## Supplementary Materials

The following are available online at https://www.mdpi.com/2072-6694/12/1/67/s1, Table S1: Baseline laboratory values in the whole case series and according to the presence or absence of TE events, Table S2: Characteristics of Thromboembolic Events occurred during treatment with Immune-Checkpoint Inhibitors, Table S3. Use of anticoagulant treatment according to patients’ baseline ECOG PS, Table S4: Baseline patients’ characteristics in the whole case series and according to antiplatelet use, Figure S1: TE-specific hazard according to smoking status, Figure S2: TE-specific hazard according to PD-L1 status, Figure S3: TE-specific hazard according to Platelet to Lymphocyte Ratio (PLR), Figure S4: TE-specific hazard according to number of disease sites, Figure S5: Overall Survival Among Patients with or without TE Events considered as a time-independent variable, Figure S6: Progression Free Survival according to Aspirin (ASA) treatment.

## Abbreviations and Acronyms

ASA	Aspirin
BMI	Body mass index
CI	Confidence interval
COX	Cyclooxygenase
COXi	COX Inhibitors
CT	Computerized tomography
CTLA-4	Cytotoxic T-lymphocyte antigen 4
ECOG PS	Eastern Cooperative Oncology Group Performance Status
HR	Hazards ratio
ICIs	Immune checkpoint inhibitors
irAEs	Immune-related adverse events
LDH	Lactate dehydrogenase
LIPI	Lung immune prognostic index
LMWH	Low molecular weight heparin
mPGES1	Human prostaglandin E synthase 1
NLR	Neutrophil to lymphocyte ratio
NSCLC	Non-small cell lung cancer
OS	Overall Survival
PD-1/PD-L1	Programmed death-1/programmed death-ligand 1
PFS	Progression-free survival
PGE2	Prostaglandin E2
PLR	Platelet to lymphocyte ratio
ROC	Receiver operating characteristic
TE	Thromboembolic
