# Supplementary material for: Is There an Interplay between Immune Checkpoint Inhibitors, Thromboprophylactic Treatments and Thromboembolic Events? Mechanisms and Impact in Non-Small Cell Lung Cancer Patients
Source: Cancers (Basel). 2019 Dec 25;12(1):67. doi: 10.3390/cancers12010067 (PMC7016680; doi:10.3390/cancers12010067)
Supplement: Supplementary file 1 [file cancers-12-00067-s001.pdf]

cancers

MDPI

Supplementary Materials: Is There an Interplay between Immune Checkpoint Inhibitors, Thromboprophylactic Treatments and Thromboembolic Events? Mechanisms and Impact in Non-Small Cell Lung Cancer Patients

Federico Nichetti, Francesca Ligorio, Emma Zattarin, Diego Signorelli, Arsela Prelaj, Claudia Proto, Giulia Galli, Antonio Marra, Giulia Apollonio, Luca Porcu, Filippo de Braud, Giuseppe Lo Russo, Roberto Ferrara and Marina Chiara Garassino

Table S1. Baseline laboratory values in the whole case series and according to the presence or absence of TE events.

Characteristic	Overall *n* = 217	Without TE *n* = 187	With TE *n* = 30
LDH			
Normal	163 (75.1)	140 (74.9)	23 (76.7)
> 480 U/L §	42 (19.4)	35 (18.7)	7 (23.3)
Leucocytes			
≥11 × 10⁹/L	45 (20.7)	39 (20.9)	6 (20.0)
<11 × 10⁹/L	170 (78.3)	146 (78.1)	24 (80)
Neutrophils, median (range)	5400 (1100–51600)	5500 (1100–51600)	5350 (2600–15600)
>8 × 10⁹/L §	171 (79.5)	147 (78.6)	24 (80.0)
≤8 × 10⁹/L	44 (20.5)	38 (20.3)	6 (20.0)
Lymphocytes, median (range)	1400 (100–7600)	1300 (100–3800)	1600 (200–7600)
Hemoglobin			
≥10 g/dl	200 (92.2)	171 (91.4)	29 (96.7)
<10 g/dl	15 (6.9)	14 (7.5)	1 (3.3)
Platelets			
≥ 350 × 10⁹/L	62 (28.6)	53 (28.3)	9 (30.0)
< 350 × 10⁹/L	153 (70.5)	132 (70.6)	21 (70.0)
PLR, median (range)	201 (27–1390)	262 (53–1390)	219 (27–760)
>181	126 (58.1)	117 (62.6)	9 (30.0)
≤181	89 (41.0)	68 (36.4)	21 (70.0)
NLR, median (range)	3.9 (0.6–38.7)	4.1 (0.6–38.7)	3.2 (1.0–33.0)
>3.2	141 (65.0)	127 (67.9)	14 (46.7)
≤3.2	74 (34.1)	58 (31.0)	16 (53.3)
Khorana Score¶			
1	122 (56.2)	105 (56.1)	17 (56.7)
2	57 (26.3)	49 (26.2)	8 (26.7)
≥3	35 (16.1)	30 (16.0)	5 (16.7)
LIPI			
Low	60 (27.6)	47 (25.1)	13 (43.3)
Intermediate	12 (51.6)	99 (52.9)	13 (43.3)
High	33 (15.2)	29 (15.5)	4 (13.3)

All blood tests were performed at INT. Data for blood cell count was not available for 2 pts. Cut-offs for hemoglobin and platelets counts were chosen according to the Khorana Score. The following parameters were calculated as follows: (a) NLR by dividing neutrophil by lymphocyte counts; (b) PLR by dividing platelet by lymphocyte counts; (c) LIPI based on NLR greater than 3 and LDH greater than institutional laboratory ULN;.(d) Khorana Score based on: lung cancer (1 point); baseline leukocyte count ≥11 × 109/L (1 point), platelet count ≥ 350 × 109/L (1 point), hemoglobin < 10 g/dL (1 point), and BMI ≥ 35kg/m² (1 point). Receiver operating characteristic (ROC) curves were used to set

the optimal threshold for NLR and PLR according to the occurrence of a TE. § reference ULN for internal laboratory. Data for LDH was not available for 12 pts. data was missing for 3 patients. Abbreviations: LDH: Lactate Dehydrogenase; LIPI: Lung Immune Prognostic Index; NLR: neutrophil to lymphocyte ratio; PLR: platelet to lymphocyte ratio; TE: thromboembolic events; ULN: upper limit of normal.

Table S2. Characteristics of Thromboembolic Events occurred during treatment with Immune-Checkpoint Inhibitors.

Patient	TE Event-Free Time (Months)	N° of Administered Cycles	Type of TE Event
1	1.4	2	PE
2	1.3	2	Portal vein thrombosis
3	1.2	3	Upper limb DVT
4	1.3	3	Cerebrovascular accident
5	1.4	3	Cerebrovascular accident
6	1.9	4	PE
7	2.6	4	Thrombosis of the abdominal aorta
8	2.5	5	Acute coronary syndrome
9	2.0	5	Thrombosis of the abdominal aorta
10	10.4	6	Jugular vein thrombosis
11	2.1	6	Lower limb DVT + PE + iliac vein thrombosis
12	7.8	9	Lower limb DVT + PE
13	6.3	9	Cerebrovascular accident
14	7.5	10	Thrombosis of the abdominal aorta
15	7.6	12	PE
16	5.8	12	Lower limb DVT + PE + Upper limb DVT
17	5.3	12	Lower limb DVT
18	10.1	13	Upper limb DVT
19	9.7	19	Cerebrovascular accident
20	18.5	20	Cerebrovascular accident
21	10.2	20	PE
22	13.6	21	PE
23	18.3	26	Lower limb DVT
24	14.0	27	Portal vein thrombosis
25	6.5	30	Cerebrovascular accident
26	20.8	39	Acute coronary syndrome
27	23.8	43	PE
28	31.4	66	Cerebrovascular accident
29	31.2	67	Cerebrovascular accident
30	33.7	71	Cerebrovascular accident
TE-EFT	7.5	1.2–33.6	
Arterial	7.0	1.3–33.6	
Venous	7.7	1.2–23.7	Log-Rank $p = 0.59$

Abbreviations: PE: pulmonary embolism; DVT: deep vein thrombosis; TE-EFT: thromboembolic event-free time.

Table S3. Use of anticoagulant treatment according to patients' baseline ECOG PS.

Anticoagulant Treatment	ECOG PS 0–1	ECOG PS ≥ 2	*p*
No	159 (80.3)	11 (57.9)	**0.05**
Yes	39 (19.7)	8 (42.1)	

Data are presented as n (%). The p value of the χ^2 test is indicated in the right column of the table. Abbreviations: ECOG PS: Eastern Cooperative Oncology Group Performance status.

Table S4. Baseline patients' characteristics in the whole case series and according to antiplatelet use.

Characteristic	Overall<br>n = 217	Antiplatelet Non-Users<br>n = 148	Antiplatelet Users<br>n = 69	*p*
Sex				
Male	136 (62.7)	84 (56.8)	52 (75.4)	**0.012**
Age, median (range), y	70 (32–90)	67 (32–88)	74 (58–90)	**<0.001**
≤65	77 (35.5)	69 (46.6)	8 (11.6)	
>65	140 (64.5)	79 (53.4)	61 (88.4)	**<0.001**
Comorbidities				
Arterial hypertension	44 (20.3)	26 (17.6)	18 (26.1)	0.20
COPD	25 (11.5)	13 (8.8)	12 (17.4)	0.11
Diabetes Mellitus	21 (9.7)	7 (4.7)	14 (20.3)	**0.001**
Previous ACS	18 (8.3)	2 (1.4)	16 (23.2	**<0.001**
Previous Stroke	9 (4.1)	3 (2.0)	6 (8.7)	**0.05**
Atrial Fibrillation	10 (4.6)	6 (4.1)	4 (5.8)	0.68
Previous venous TE events	37 (17.7)	19 (12.8)	18 (26.1)	**0.03**
Use of LMWH	47 (21.7)	40 (27)	7 (10.1)	**0.001**

The *p* value is indicated in bold numbers when statistically significant. Data are presented as *n* (%) except where otherwise noted. The *p* value of the χ^2 and MWW test assessing the association between each characteristic and ASA use is indicated in the right column of the table. The *p* value of the test is indicated in bold numbers when statistically significant. Abbreviations: ACS: acute coronary syndrome; LMWH: Low Molecular Weight Heparin; TE events: thromboembolic events

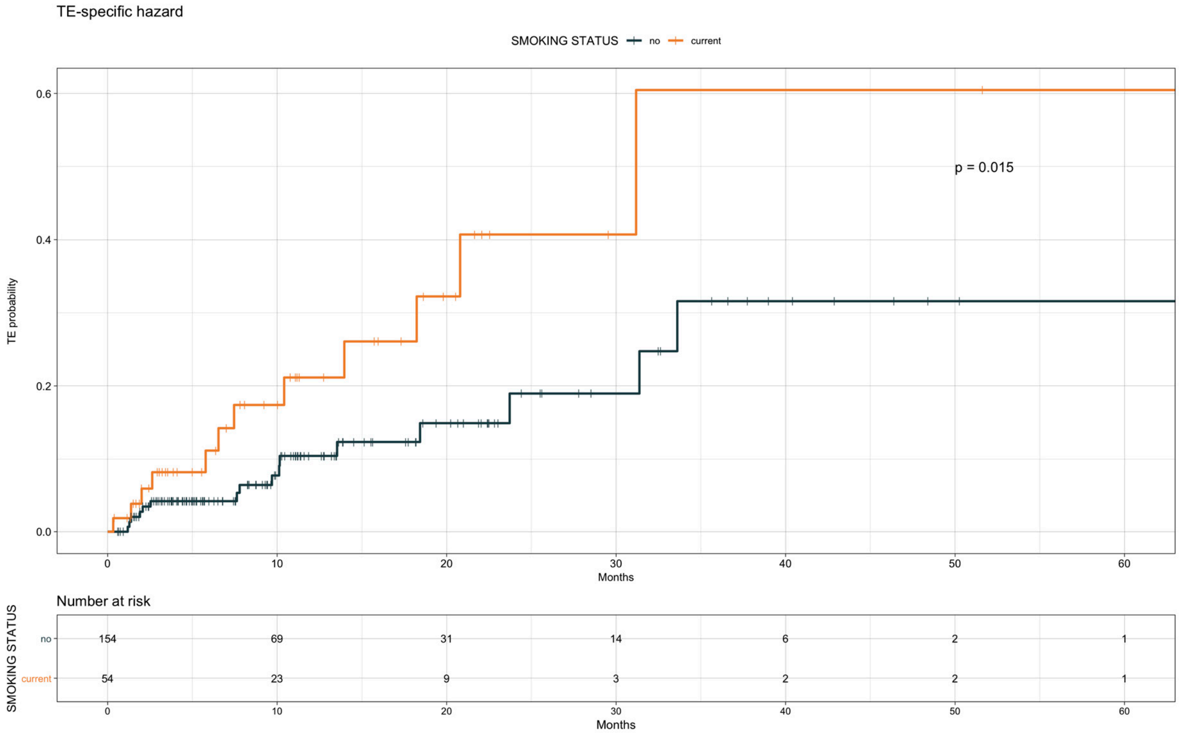

Figure S1. TE-specific hazard according to smoking status.

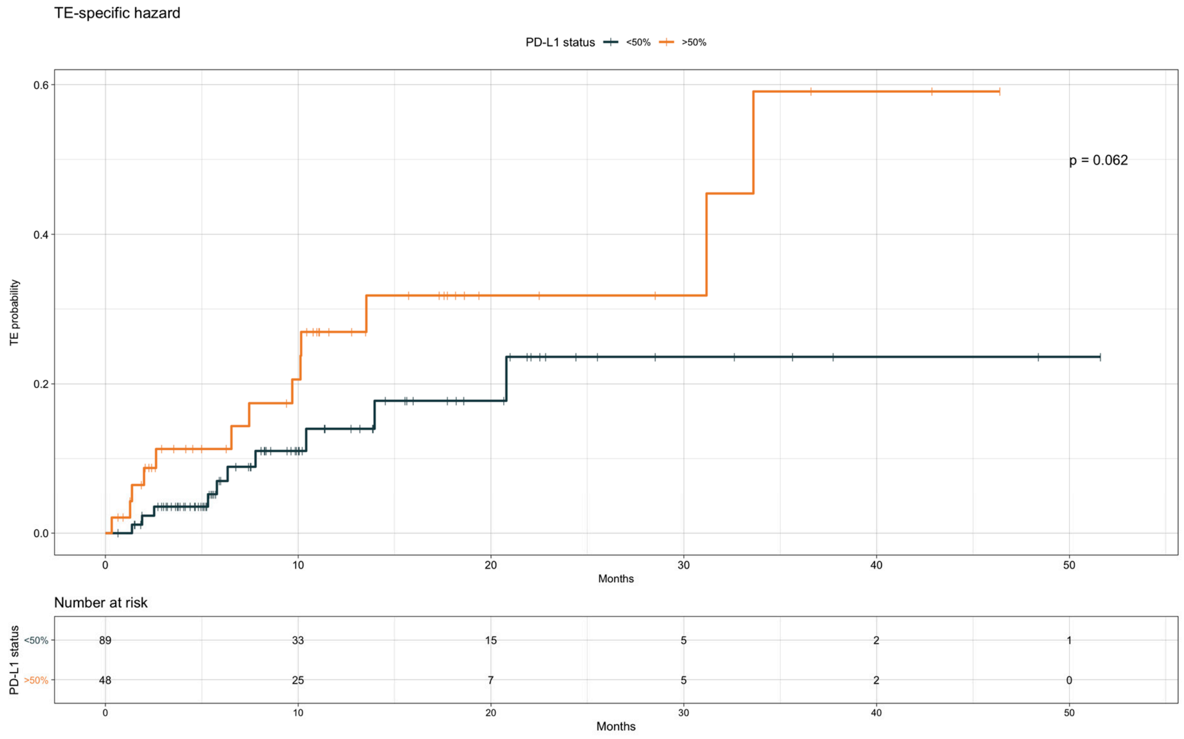

Figure S2. TE-specific hazard according to PD-L1 status.

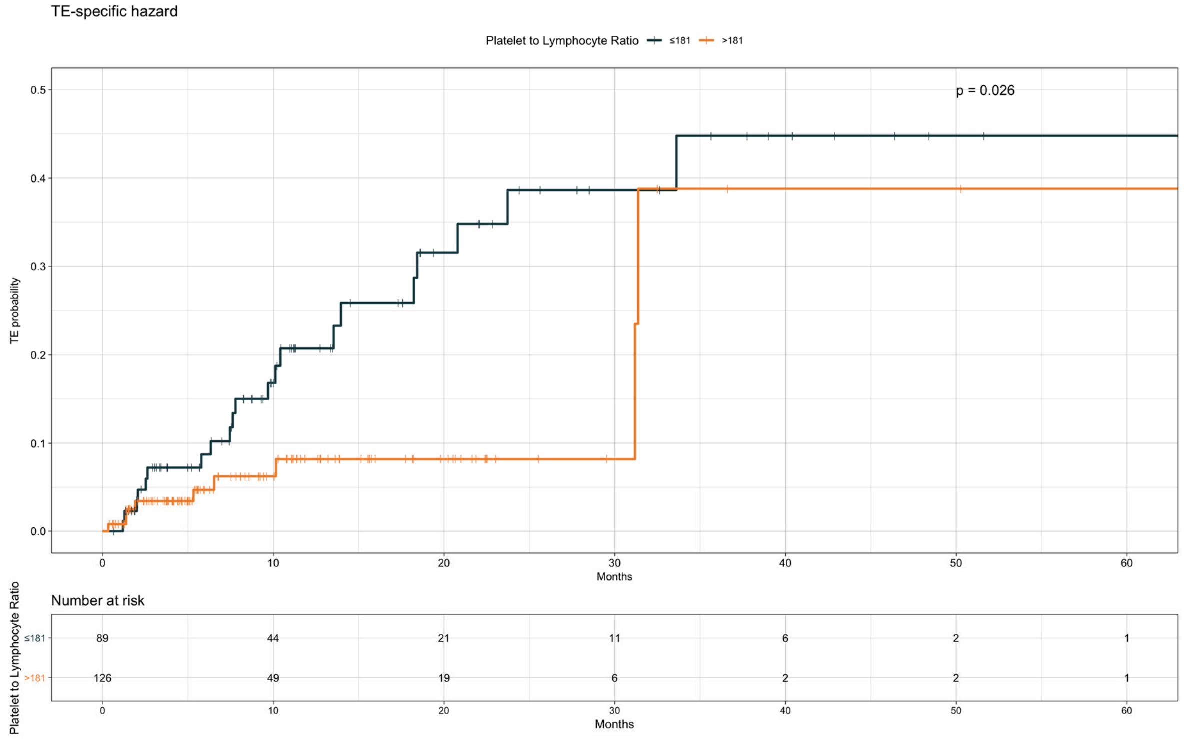

Figure S3. TE-specific hazard according to Platelet to Lymphocyte Ratio (PLR).

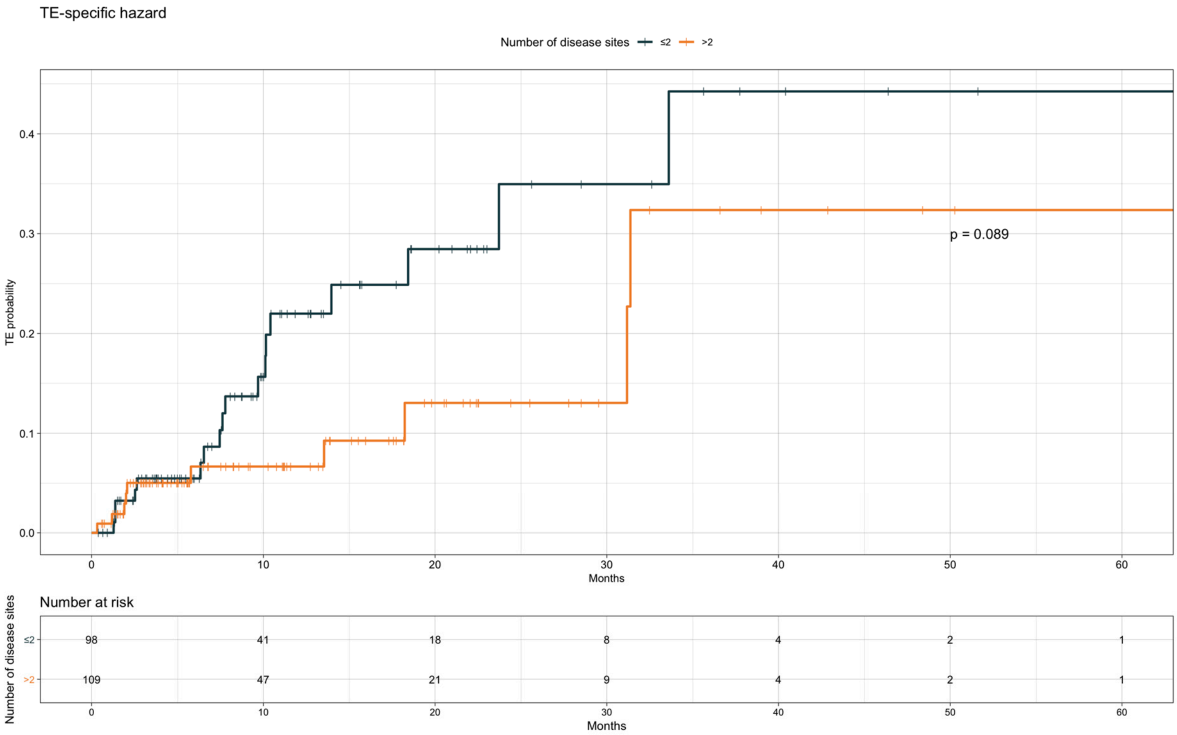

Figure S4. TE-specific hazard according to number of disease sites.

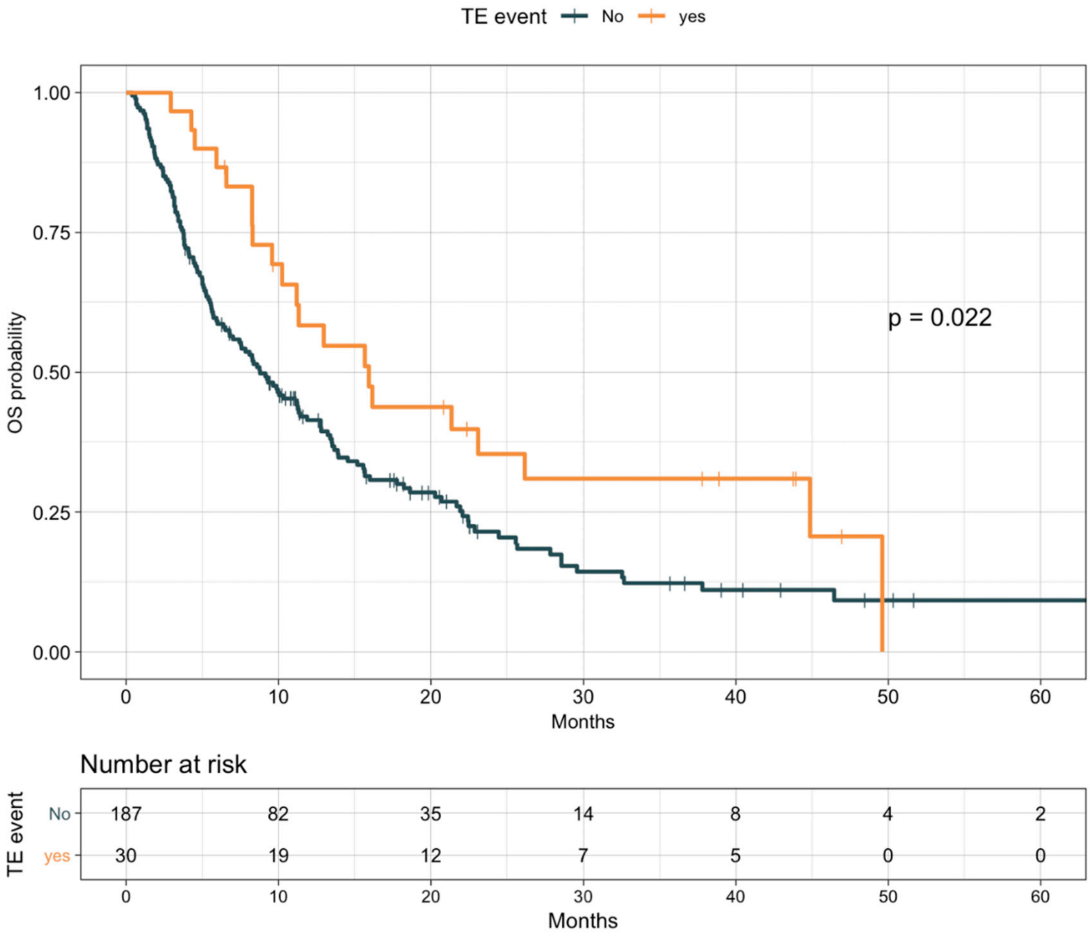

Figure S5. Overall Survival Among Patients with or without TE Events considered as a time-independent variable.

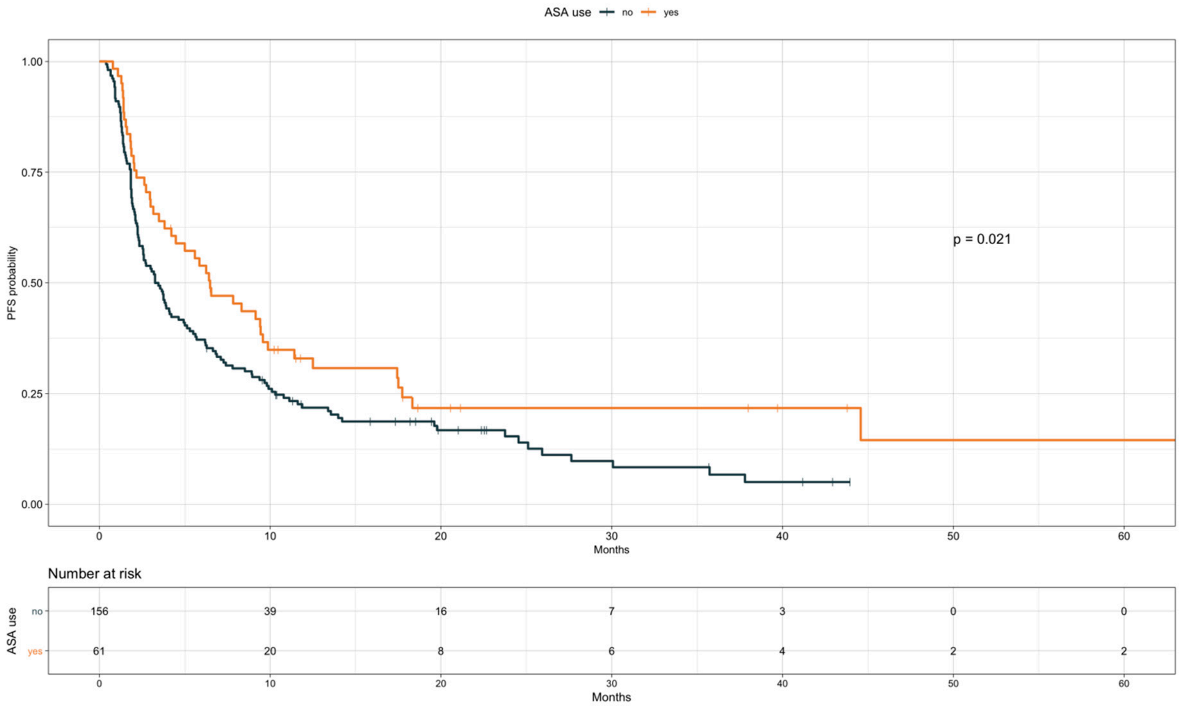

Figure S6. Progression Free Survival according to Aspirin (ASA) treatment.

© 2019 by the authors. Licensee MDPI, Basel, Switzerland. This article is an open access article distributed under the terms and conditions of the Creative Commons Attribution (CC BY) license (http://creativecommons.org/licenses/by/4.0/).
